# Function-Based Tractography of the Language Network Correlates with Aphasia in Patients with Language-Eloquent Glioblastoma

**DOI:** 10.3390/brainsci10070412

**Published:** 2020-07-01

**Authors:** Haosu Zhang, Severin Schramm, Axel Schröder, Claus Zimmer, Bernhard Meyer, Sandro M. Krieg, Nico Sollmann

**Affiliations:** 1 Department of Neurosurgery, Klinikum rechts der Isar, Technische Universität München, Ismaninger Str. 22, 81675 Munich, Germany; Haosu.Zhang@gmail.com (H.Z.); Schrammseverin@gmail.com (S.S.); Axel.Schroeder@tum.de (A.S.); Bernhard.Meyer@tum.de (B.M.); Sandro.Krieg@tum.de (S.M.K.); 2 Department of Diagnostic and Interventional Neuroradiology, Klinikum rechts der Isar, Technische Universität München, Ismaninger Str. 22, 81675 Munich, Germany; Claus.Zimmer@tum.de; 3 TUM-Neuroimaging Center, Klinikum rechts der Isar, Technische Universität München, 81675 Munich, Germany

**Keywords:** brain stimulation, fiber tractography, glioblastoma multiforme, gray matter, language mapping, navigated transcranial magnetic stimulation

## Abstract

To date, the structural characteristics that distinguish language-involved from non-involved cortical areas are largely unclear. Particularly in patients suffering from language-eloquent brain tumors, reliable mapping of the cortico-subcortical language network is of high clinical importance to prepare and guide safe tumor resection. To investigate differences in structural characteristics between language-positive and language-negative areas, 20 patients (mean age: 63.2 ± 12.9 years, 16 males) diagnosed with language-eloquent left-hemispheric glioblastoma multiforme (GBM) underwent preoperative language mapping by navigated transcranial magnetic stimulation (nTMS) and nTMS-based diffusion tensor imaging fiber tracking (DTI FT). The number of language-positive and language-negative points as well as the gray matter intensity (GMI), normalized volumes of U-fibers, interhemispheric fibers, and fibers projecting to the cerebellum were assessed and compared between language-positive and language-negative nTMS mappings and set in correlation with aphasia grades. We found significantly lower GMI for language-positive nTMS points (5.7 ± 1.7 versus 7.1 ± 1.6, *p* = 0.0121). Furthermore, language-positive nTMS points were characterized by an enhanced connectivity profile, i.e., these points showed a significantly higher ratio in volumes for U-fibers (*p* ≤ 0.0056), interhemispheric fibers (*p* = 0.0494), and fibers projecting to the cerebellum (*p* = 0.0094). The number of language-positive nTMS points (R ≥ 0.4854, *p* ≤ 0.0300) as well as the ratio in volumes for U-fibers (R ≤ −0.4899, *p* ≤ 0.0283) were significantly associated with aphasia grades, as assessed pre- or postoperatively and during follow-up examinations. In conclusion, this study provides evidence for structural differences on cortical and subcortical levels between language-positive and language-negative areas, as detected by nTMS language mapping. The results may further increase confidence in the technique of nTMS language mapping and nTMS-based tractography in the direct clinical setting. Future studies may confirm our results in larger cohorts and may expand the findings to patients with other tumor entities than GBM.

## 1. Introduction

Resection of intracranial glioma aims at a maximum extent of resection, which should ideally be achieved without causing surgery-related functional deficits that could severely reduce the patients’ quality of life [1,2,3,4]. To establish maximum resection whilst avoiding functional decline as far as possible, several pre- and intraoperative techniques have been developed to assist in neurosurgical planning and resection guidance [4,5,6,7]. For the intraoperative setting, cortical and subcortical direct electrical stimulation (DES) serves as the current gold-standard method [8,9,10]. Regarding the preoperative setting, navigated transcranial magnetic stimulation (nTMS) has found its way into neurosurgery over the last decade [6,11,12].

The technique of nTMS has lately been used to conduct language mappings in patients suffering from language-eloquent glioma or other entities of brain tumors [13,14,15,16]. Furthermore, it has been combined with diffusion tensor imaging (DTI) derived from preoperative magnetic resonance imaging (MRI) to provide spatially resolved maps that visualize language-related structures [17,18,19,20,21,22,23,24,25]. Integration into clinical routine and the perioperative workflow is seamless, and the approach is currently regarded as a valuable adjunct to intraoperative DES [14,15]. However, in direct comparison to intraoperative DES, nTMS language mapping has shown a rather low specificity of 23.8% and a positive predictive value of 35.6% [13]. The mere additional use of nTMS-based DTI fiber tracking (DTI FT) did not improve the identification of DES-positive language areas during awake surgery [24]. Nevertheless, the visualization of the subcortical language network becomes possible purely based on functional data by using nTMS-based DTI FT, which has shown high potential for surgical planning, resection guidance, and risk assessment in patients with language-eloquent lesions [17,18,19,20,21,22,23,24,25]. However, explorations of the differences between language-positive and language-negative nTMS mappings are still largely missing, which is one reason contributing to the lack of understanding of the comparatively low specificity of nTMS language mapping in relation to intraoperative DES. Further insights may lead to a better definition of the role of nTMS in the neurosurgical setting and to improved understanding of nTMS characteristics.

The cortico-subcortical network behind human language function is complex [26,27,28,29]. On the cortical level, differences in gray matter (GM) distribution could probably differentiate between language-related and non-related areas or, at least, between highly and less involved areas. In this regard, previous research has demonstrated that the degree to which language is lateralized to one of the hemispheres is positively predicted by the degree to which GM is lateralized on a voxel-by-voxel basis [30]. In subjects with dyslexia, a GM deficit involving a fronto-temporal network important for phonological processing was revealed, whereas region-specific increases in GM volume are possible for developmental language disorders as a result of compensatory mechanisms [31,32]. On the subcortical level, differences in connectivity profiles between areas depending on language involvement seem likely. Besides major language-related white matter tracts known to be involved in language processing, various short or long interconnecting fibers, such as short association fibers (commonly referred to as arcuate fibers or U-fibers), interhemispheric transcallosal fibers, and fibers projecting to the cerebellum play a role [28,29,33,34]. 

Against this background, this study’s objective is to systematically explore characteristics of language-positive structures according to nTMS language mapping and nTMS-based DTI FT in direct comparison to language-negative counterparts. We hypothesize that (1) language-positive cortical areas may show different focal GM intensity (GMI, as a potential expression of higher GM density as a correlate of increased functional involvement), and that (2) language-positive cortical areas show a different connectivity profile (higher volumes of U-fibers, transcallosal fibers, and fibers projecting to the cerebellum) when compared to language-negative cortical areas.

## 2. Materials and Methods

### 2.1. Ethics

The current study is in accordance with the Declaration of Helsinki and its later amendments and has been approved by the local institutional review board (registration numbers: 2793/10, 5811/13, 223/14, and 336/17). Written informed consent was obtained from all patients enrolled in this study.

### 2.2. Patients and Study Inclusion

This study is a post-hoc analysis including patients of our prospectively enrolled cohort that underwent language mapping by nTMS and nTMS-based DTI FT of language-related fiber tracts prior to resection of a brain tumor. The following inclusion criteria were defined for the present study:

(1) Written informed consent,
(2) Age above 18 years,
(3) German as first language,
(4) Left-hemispheric perisylvian tumor location (MRI suggesting infiltration and/or compression of anatomically suspected cortical language-eloquent areas and/or suspected close proximity to subcortical language-related pathways),
(5) Availability of preoperative 3-Tesla MRI, including a DTI sequence with 32 diffusion directions,
(6) Clinical indication for preoperative nTMS language mapping and nTMS-based DTI FT,
(7) Surgery for tumor resection and final diagnosis of a glioblastoma multiforme (GBM) according to histopathological examination (based on tumor tissue probes taken during resection), and
(8) Follow-up time of at least 3 months after surgery.

The exclusion criteria were defined as follows:

(1) Multilingual background (regular input in more than one language between birth and adolescence),
(2) Neurological or psychiatric diseases (except for the diagnosis of a GBM), and
(3) Aphasia to a degree not allowing for preoperative language mapping by nTMS.

### 2.3. Clinical Examination 

A standardized assessment of sensory function, coordination, muscle strength, and cranial nerve function was performed as part of the initial clinical examination. In particular, the language status was evaluated by a neuropsychologist using the Aachen Aphasia Test and by categorizing language deficits on a four-point scale [14,35,36,37]. In detail, four grades were established, which were no deficit (grade 0), mild deficit (grade 1: normal language comprehension and/or conversational language with slight amnesic aphasia, adequate communication ability), medium deficit (grade 2: minor disruption of language comprehension and/or conversational language, adequate communication ability), and severe deficit (grade 3: major disruption of language comprehension and/or conversational language, clear impairment of communication ability) [14,35,36,37]. Furthermore, handedness was assessed by the Edinburgh Handedness Inventory (EHI) [38]. The clinical examinations including detailed assessments of language function were repeated postoperatively and during the routine follow-up examinations. 

### 2.4. Cranial Magnetic Resonance Imaging 

Imaging was performed on a 3-Tesla MRI scanner using a 32-channel head coil (Achieva dStream or Ingenia; Philips Healthcare, Best, The Netherlands). As part of a standardized, multi-sequence imaging protocol for brain tumors, a three-dimensional fluid attenuated inversion recovery (FLAIR) sequence (repetition time (TR)/echo time (TE): 4800/277 ms, 1 mm^3^ isovoxel covering the whole head), three-dimensional T1-weighted gradient echo sequence (TR/TE: 9/4 ms, 1 mm^3^ isovoxel covering the whole head) without and with application of an intravenous contrast agent (Dotagraf, Jenapharm GmbH & Co. KG, Jena, Germany), and a DTI sequence (TR/TE 5000/78 ms, voxel size of 2 × 2 × 2 mm^3^, 32 diffusion gradient directions) were acquired preoperatively and were used for nTMS language mapping (contrast-enhanced T1-weighted sequences) and nTMS-based DTI FT (contrast-enhanced T1-weighted, DTI, and FLAIR sequences). 

Postoperative (within the first 48 h after surgery) and follow-up imaging were performed using the same imaging protocol. In postoperative MRI, special attention was paid to the assessment of residual tumor tissue or achieved gross total resection (GTR) as well as perioperative bleeding or ischemia. 

### 2.5. Language Mapping by Navigated Transcranial Magnetic Stimulation

#### 2.5.1. Mapping Procedure

Preoperative language mapping by nTMS of the tumor-affected left hemisphere (LH) was carried out during the days before scheduled surgery for tumor resection using a Nexstim eXimia NBS system (version 4.3; Nexstim Plc, Helsinki, Finland) [11,14,15,39,40]. Language mappings were conducted in German as the native language of all enrolled patients. 

After initial co-registration of the patient’s head and the contrast-enhanced T1-weighted image dataset, pictures of common objects were presented to the patient in the context of two baseline trials (presentation of objects without simultaneous stimulation) [13,40,41,42,43,44]. The objects shown were part of a standardized object-naming task, and the purpose of baseline assessment was to systematically discard objects that did not elicit a quick and fluent response and to familiarize the patients with the task and setup. The remaining stack of objects that were named correctly and fluently was then shown under stimulation of up to 46 target points that had been placed on the LH, with each point being stimulated six times in total (stimulation intensity: 100% of the individual resting motor threshold, stimulation frequency: 5 Hz/5 pulses) [13,40,42,43]. 

**Figure 1:**
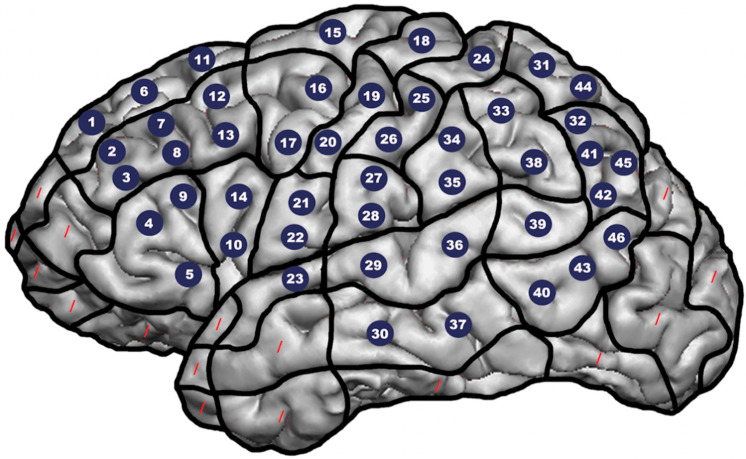
Cortical parcellation system (CPS). The tumor-affected left hemisphere (LH) was mapped by navigated transcranial magnetic stimulation (nTMS) with respect to 46 target points that were placed in relation to the CPS (reduced numbers of target points were accepted in patients with large tumor masses, which hampered placement of the total amount of 46 target points). The points were stimulated six times in total each, with a stimulation intensity of 100% of the individual resting motor threshold and a stimulation frequency of 5 Hz/5 pulses. Numbers in circles schematically represent the stimulation targets on a standardized brain template, red dashes mark regions that were not subject to nTMS.

The 46 target points for stimulation were tagged with close respect to the cortical parcellation system (CPS), thus covering almost the whole LH except for polar, occipital, and inferior temporal regions, similar to previous studies on nTMS language mapping (Figure 1) [13,42,43]. Exclusion was due to considerable muscle activation and/or discomfort that can be observed following stimulation of these particular regions. Furthermore, in patients showing large tumor masses, the number of 46 target points for stimulation needed to be reduced according to individual cortical architecture given destructions and derangements caused by such lesions. The picture-to-trigger interval during stimulation was set as 0 ms by default [45,46]. The patients’ performance during object naming was video-recorded for later evaluation to detect and categorize errors elicited by targeted nTMS [41,44].

#### 2.5.2. Mapping Evaluation

Video data of the nTMS language mappings were systematically searched for naming errors of different categories after the mapping procedure. No responses, performance errors, hesitations, neologisms, phonological paraphasias, and semantic paraphasias were defined and considered during evaluation [13,41,42,43,44]. The stimulation points that elicited any naming error except hesitations (no responses, performance errors, neologisms, phonological paraphasias, and semantic paraphasias together) were considered as language-positive nTMS points. Correspondingly, stimulation points that did not elicit a naming error were defined as language-negative nTMS points. 

Per patient, two export datasets were then generated, which included the language-positive nTMS points of the LH and the language-negative nTMS points of the LH, respectively. Each export file was in Digital Imaging and Communications in Medicine (DICOM) format and included the nose and ears as anatomical landmarks together with the stimulation points. Furthermore, in each patient, CPS regions were counted as language-positive CPS regions when they contained at least one language-positive nTMS point, while CPS regions without language-positive nTMS points were considered as language-negative CPS regions. CPS regions that were not stimulated (polar, occipital, and inferior temporal regions) were not considered. The total number of language-positive CPS regions was recorded as N_p_, and the number of language-negative CPS regions as N_n_. 

### 2.6. Tractography Based on Navigated Transcranial Magnetic Stimulation

Deterministic fiber tractography was based on nTMS language mapping data without additional anatomical seeding (Brainlab Elements, version 3.1.0; Brainlab AG, Munich, Germany). Both export datasets were first transferred to an external server and then auto-fused with the MRI sequences of the respective patient, using manual correction in case of registration misalignments and eddy current correction for DTI data. The datasets containing language-positive or language-negative nTMS points were separately defined as three-dimensional objects, followed by generation of regions of interest (ROIs) out of these objects by adding a rim of 5 mm to each stimulation point [14,15,17,18,19]. 

An individual fractional anisotropy (FA) value, the fractional anisotropy threshold (FAT), was defined separately in each patient for nTMS-based DTI FT using the ROI constituted of language-positive or language-negative nTMS points, which was determined by setting angulation to 90°, the minimum fiber length (FL) to 30 mm, and increasing the FA stepwise until no fibers were displayed, followed by decreasing the FA by 0.01, thus visualizing a minimum fiber course. The corresponding FA value was defined as 100% FAT. A similar approach using a higher minimum FL for FAT determination has been used previously for nTMS-based DTI FT [17,19,47].

After FAT definition, nTMS-based DTI FT was carried out separately with the ROIs of language-positive and language-negative nTMS points using the respective FA values for 100% FAT, 75% FAT, 50% FAT, and 25% FAT, and a minimum FL of 30 mm as well as 3 mm, respectively. The values of 30 and 3 mm were considered for the minimum FL due to the characteristic length of U-fibers that have been described to be typically in this range [48].

### 2.7. Data Analyses

**Figure 2:**
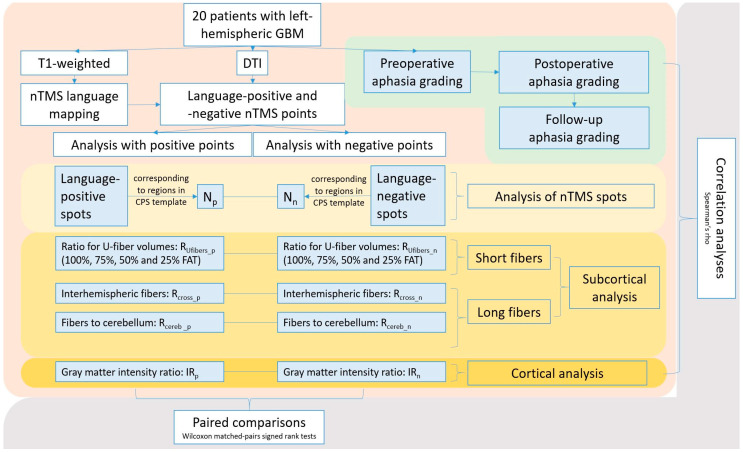
Overview of procedures and analyses. This scheme summarizes the study’s steps of analyses, which were performed based on data derived from language mapping by navigated transcranial magnetic stimulation (nTMS) and nTMS-based diffusion tensor imaging fiber tracking (DTI FT). Language-positive and language-negative nTMS points were used separately for the different analyses. The study cohort included 20 patients with left-hemispheric glioblastoma multiforme (GBM).

The data analyses were divided into two parts (Figure 2). The first part focused on the cortical level to investigate differences in GMI between language-positive and language-negative nTMS points (Figure 2). The second part focused on the subcortical level to assess differences in fiber tractography between nTMS-based DTI FT using language-positive or language-negative nTMS points as ROIs by means of investigating short fibers for short-distance connections (U-fibers), long fibers projecting to the contralateral hemisphere (Cross-F), and fibers projecting to the cerebellum (Cereb-F; Figure 2). 

#### 2.7.1. Gray Matter Intensity 

**Figure 3:**
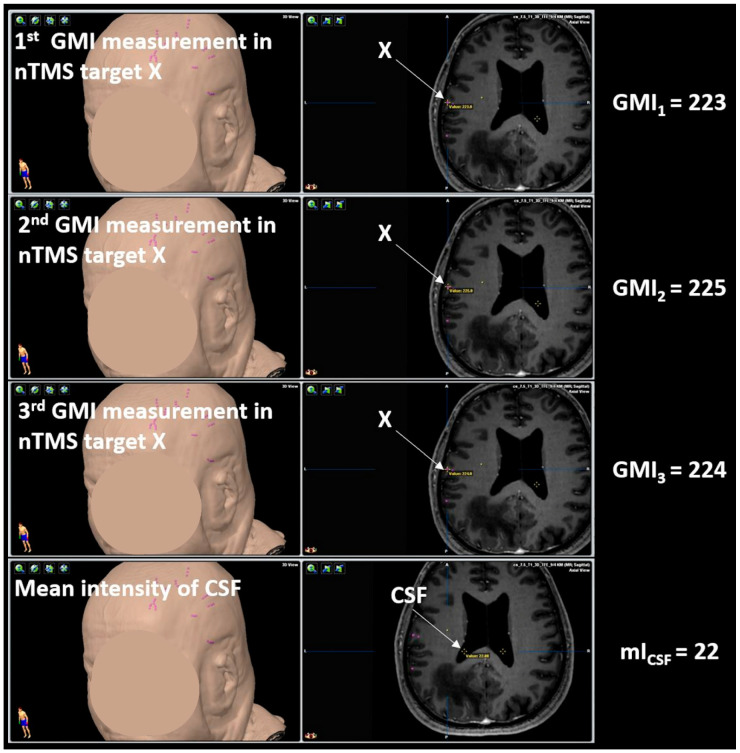
Analysis of gray matter intensity (GMI). The GMI of language-positive or language-negative spots, determined by language mapping using navigated transcranial magnetic stimulation (nTMS), was measured in T1-weighted sequences. Three pixels were randomly selected within a stimulation spot and used to calculate a mean GMI for language-positive nTMS spots (mGMI_p_) and language-negative nTMS spots (mGMI_n_). The signal intensity ratio (IR) was then calculated by dividing the mGMI_p_ or mGMI_n_ by the mean intensity of cerebrospinal fluid (mI_csf_).

Using the T1-weighted sequences co-registered to language-positive or language-negative nTMS points, the GMI was measured per stimulation point (iPlan Net server, version 3.0.1; Brainlab AG, Munich, Germany). Three pixels were randomly selected within a stimulation point and used for GMI measurements to represent the GMI per stimulation point by averaging the three values obtained (Figure 3). The mean GMI of language-positive nTMS points was recorded as mGMI_p_, and the mean GMI of language-negative nTMS points as mGMI_n_ in each patient (Figure 3). Then, intensity extraction was also conducted for the cerebrospinal fluid (CSF) using three randomly selected pixels in the lateral ventricles, and the mean was recorded as mI_csf_ to obtain an internal control value (Figure 3). The signal intensity ratio (IR) was calculated as follows [49]:

(1) $$IR_{p}=\frac{mGMI_{p}}{mI_{csf}}$$

(2) $$IR_{n}=\frac{mGMI_{n}}{mI_{csf}}$$

#### 2.7.2. U-Fibers 

**Figure 4:**
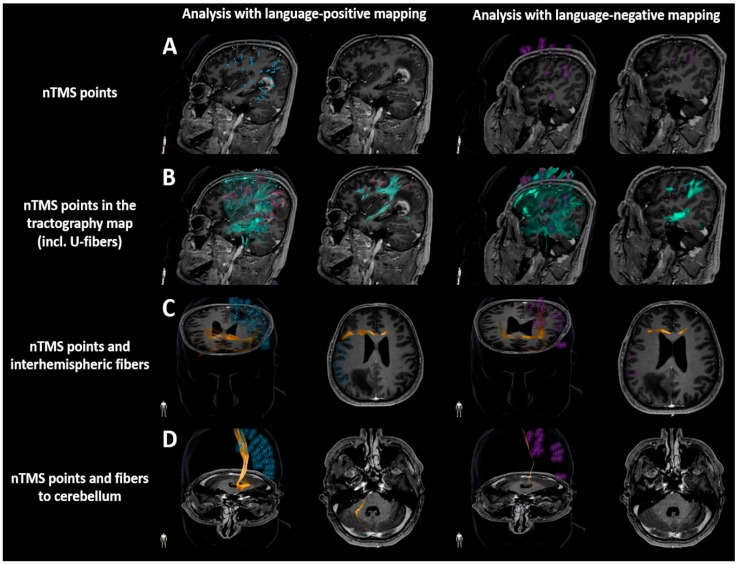
Analysis of subcortical fiber tracts. Tractography maps were generated based on language mapping data derived from navigated transcranial magnetic stimulation (nTMS), using the language-positive nTMS spots and language-negative nTMS spots as separate regions of interest (ROIs). Row (**A**) visualizes the language-positive nTMS points (blue) and language-negative nTMS points (purple) in an exemplary patient case with a left-hemispheric contrast-enhancing tumor with temporo-parietal location. Row (**B**) illustrates the complete picture of tractography (green fibers) with special emphasis on U-fibers (purple fibers). Row (**C**) depicts interhemispheric fiber courses (orange fibers) crossing the midline via the corpus callosum. Row (**D**) visualizes fibers projecting to the cerebellum (orange fibers).

Using nTMS-based DTI FT with the different FAT levels and a minimum FL of 30 and 3 mm, volumes of U-fibers were measured for fiber tractography considering language-positive or language-negative nTMS points as ROIs, respectively (Brainlab Elements, version 3.1.0; Brainlab AG, Munich, Germany; Figure 4). From assessing the difference in volumes between fibers with a minimum FL of 30 mm and fibers with a minimum FL of 3 mm, the volume of U-fibers was obtained (Figure 4). In this context, U-fibers are considered short association fibers, which connect cortical regions between adjacent gyri and typically have a length in the range of 3 to 30 mm [48]. 

A higher number of nTMS points considered during tractography should lead to more fibers, which was corrected for by dividing the fiber volume by N_p_ or N_n_, resulting in the ratios R_Ufibers_*p*_ and R_Ufibers_n_ for fibers with a length of 3 to 30 mm. The ratios in R_Ufibers_*p*_ and R_Ufibers_n_ were calculated as follows:

(3) $$R_{Ufibers{\_}_{p}}=\frac{V_{p\left(3\right)}-V_{p\left(30\right)}}{N_{p}}$$

(4) $$R_{Ufibers\_n}=\frac{V_{n\left(3\right)}-V_{n\left(30\right)}}{N_{n}}$$

V_p(30)_ indicates the volume of fibers with a minimum FL of 30 mm, as derived from nTMS-based DTI FT using the ROI of language-positive nTMS points, whereas V_p(3)_ represents the respective volume for a minimum FL of 3 mm. Analogously, V_n(30)_ indicates the volume of fibers with a minimum FL of 30 mm, as derived from nTMS-based DTI FT that considers the ROI of language-negative nTMS points, whereas V_n(3)_ represents the respective volume for a minimum FL of 3 mm.

#### 2.7.3. Interhemispheric Fibers and Fibers Projecting to the Cerebellum

The highest percentage of patients presenting Cross-F or Cereb-F according to visual image inspection of tractography maps was achieved for 25% FAT, which was then taken as the adjustment used for evaluation of Cross-F and Cereb-F volumes, together with a minimum FL of 30 mm derived from language-positive or language-negative nTMS points as ROIs, respectively (Brainlab Elements, version 3.1.0; Brainlab AG, Munich, Germany; Figure 4).

The volumes of the Cross-F and Cereb-F for nTMS-based DTI FT with 25% FAT were recorded as V_cross_*p*_ and V_cereb_*p*_ when derived from nTMS-based DTI FT using the ROI of language-positive nTMS points, whereas V_cross_n_ and V_cereb_n_ represent the Cross-F and Cereb-F volumes for nTMS-based DTI FT conducted with language-negative nTMS points as the ROI. The ratios R_cross_*p*_ and R_cereb_*p*_ and R_cross_n_ and R_cereb_n_ were calculated as follows:

(5) $$R_{cross\_p}=\frac{V_{\mathrm{cross}\_p}}{N_{p}}\;\mathrm{and}\;R_{cereb\_p}=\frac{V_{\mathrm{cereb}\_p}}{N_{p}}$$

(6) $$R_{cross\_n}=\frac{V_{\mathrm{cross}\_n}}{N_{n}}\;\mathrm{and}\;R_{cereb\_n}=\frac{V_{\mathrm{cereb}\_n}}{N_{n}}$$

### 2.8. Statistical Analyses

GraphPad Prism (version 6.04; GraphPad Software Inc., La Jolla, CA, USA) was used for statistical data analyses and generation of graphs. Descriptive statistics using relative and absolute frequencies or mean, standard deviation (SD), and ranges were calculated for demographics and characteristics of language mapping and tractography. The Shapiro–Wilk normality test was used to assess the distribution of data, which indicated a non-Gaussian distribution for the majority of data. 

To investigate differences between N_p_ and N_n_, IR_p_ and IR_n_, R_Ufibers_*p*_ and R_Ufibers_n_ (separately for nTMS-based DTI FT with 100% FAT, 75% FAT, 50% FAT, and 25% FAT), R_cross_*p*_ and R_cross_n_, and R_cereb_*p*_ and R_cereb_n_, Wilcoxon matched-pairs signed rank tests were performed. Furthermore, correlation analyses computing Spearman’s rho were performed between N_p_, IR_p_, R_Ufibers_*p*_, R_cross_*p*_, and R_cereb_*p*_ as well as, analogously, between N_n_, IR_n_, R_Ufibers_n_, R_cross_n_, and R_cereb_n_ and the status of preoperative, postoperative, and follow-up aphasia, considering the four grades as derived from language function assessments at different time points. The correlation analyses were adjusted for multiple testing using the Benjamini–Hochberg procedure with a false discovery rate of 25%.

## 3. Results

### 3.1. Cohort Characteristics 

Twenty patients (mean age: 63.2 ± 12.9 years, age range: 20.3–80.8 years, 4 females and 16 males, 16 right-handers according to EHI scores) were included, all diagnosed with a left-hemispheric GBM according to histopathological evaluation (Table 1). Ten patients (50%) showed preoperative aphasia, whereas eight patients (40%) showed aphasia during follow-up examinations three months after tumor resection. GTR according to postoperative MRI was achieved in 10 patients (50%).

### 3.2. Comparison between Language-Positive and Language-Negative Mapping and Tractography

Language mapping of the tumor-affected LH and nTMS-based DTI FT was possible in all enrolled patients. None of the patients showed adverse events in the course of stimulation.

There were statistically significant differences in almost all measures between mapping or tractography using language-positive or language-negative nTMS points as ROIs, respectively (Table 2). In detail, patients showed a higher mean number of language-negative nTMS points (*p* = 0.0026), whereas the IR of these points was elevated on average in comparison to language-positive nTMS points (*p* = 0.0121; Table 2). The ratios for U-fiber volumes as well as for long fibers projecting to the contralateral hemisphere and fibers projecting to the cerebellum were higher for tractography using language-positive nTMS points (*p* ≤ 0.0494; Table 2). 

### 3.3. Associations with Aphasia Grading

For language-positive nTMS points, statistically significant positive correlations were revealed between their absolute frequency and aphasia for the preoperative (R = 0.4919, *p* = 0.0276), postoperative (R = 0.6183, *p* = 0.0037), and follow-up status (R = 0.4854, *p* = 0.0300; Table 3). The higher the number of language-positive nTMS points of the tumor-affected LH, the higher the aphasia grade. Furthermore, statistically significant negative correlations were observed between the ratio of U-fiber volumes (considering tractography with 100% FAT) and aphasia for the postoperative (R = −0.6102, *p* = 0.0043) as well as follow-up status (R = −0.4899, *p* = 0.0283; Table 3). Thus, the lower this ratio was, the higher the aphasia grade. 

Regarding language-negative nTMS points, statistically significant negative correlations were revealed between their absolute frequency and aphasia for postoperative (R = −0.6097, *p* = 0.0043) and follow-up examinations (R = −0.4741, *p* = 0.0347; Table 3). Hence, the higher the number of language-negative nTMS points of the tumor-affected LH, the lower the aphasia grade.

## 4. Discussion

This study investigated the difference between language-positive and language-negative mappings as derived from presurgical nTMS and nTMS-based DTI FT in patients harboring supratentorial GBMs. There are three main results that can be taken from our analyses. First, regarding the cortical level, a significantly lower GMI was revealed for language-positive nTMS points compared to language-negative counterparts. Second, on the subcortical level, language-positive areas were characterized by an increased connectivity profile, i.e., such areas showed a significantly higher ratio in volumes for U-fibers, interhemispheric fibers, and fibers projecting to the cerebellum. Third, the number of language-positive nTMS points as well as the ratio in volumes for U-fibers were significantly associated with aphasia grading as derived from assessments at different time points. 

### 4.1. Gray Matter Intensity

While there is evidence for alterations in GM distribution and volume related to language function, the role of the GMI to characterize language-involved areas has not been investigated to the authors’ knowledge. Specifically, previous research has detected that language lateralization is predicted by the degree of GM lateralization [30]. Further, subjects diagnosed with dyslexia showed GM deficits, but the GM volume, however, can be subject to changes following training interventions in dyslexic children [31,50]. On the contrary, region-specific increases in GM volume have also been revealed for developmental language disorders, which might be interpreted as a result of compensatory mechanisms [31,32]. In the present study, lower GMI in T1-weighted sequences was revealed for language-positive nTMS points when compared to language-negative spots. This may probably reflect a sign of higher GM density, potentially suggesting increased functional involvement. Yet, this remains speculative until further studies using a similar setup can confirm our findings. For the present study, whether potentially increased functional involvement is due to higher intrinsic contribution of such areas to language function or related to compensatory mechanisms for language function at risk in our sample remains beyond the scope of investigation. However, the findings for GMI may provide a direct link between a functionally language-related area as mapped by nTMS and structural cortical characteristics. 

### 4.2. White Matter Tractography

The ratios for fiber volumes of U-fibers, transcallosal fibers, and fibers coursing to the cerebellum were higher for language-positive areas compared to language-negative counterparts. Thus, language-related spots seem to be characterized by an enhanced connectivity profile, which exists focally for short fibers connecting cortical regions between adjacent gyri (increased R_Ufibers_) as well as more remotely for long connecting fibers (increased R_cross_ and R_cereb_). This may reflect either higher a-priori involvement in the cortico-subcortical language network, compensatory mechanisms in light of language function at risk in brain tumor patients, or a mixed picture of both. While the specificity of nTMS language mapping in comparison to intraoperative DES has shown to be comparatively low with 23.8%, the technique’s negative predictive value was 83.9%, implicating that language-negative nTMS points are also mostly negative in intraoperative DES mapping [13]. The significantly lower connectivity profile of language-negative nTMS spots as revealed by the present study may resemble the “truly” absent or less involved character of these spots. Furthermore, a previous study on nTMS-based DTI FT revealed that there is a higher likelihood of subcortical connections with language-positive nTMS spots as compared to language-negative ones, with true-positive connections (connections for positive spots) being visualized up to four-fold more frequently than false-positive connections (connections for negative spots) [21]. The clear difference in this likelihood could reflect high reliability of nTMS-based DTI FT for the purpose of tracking parts of the human language network [21]. Likewise, the enhanced connectivity profile to adjacent gyri as well as the contralateral hemisphere and cerebellum may serve as a surrogate of good reliability. 

### 4.3. Associations with Aphasia

The number of language-positive nTMS points was significantly associated with aphasia grading. The higher the aphasia grading is (i.e., the more severe language impairment is), the higher the number of language-positive nTMS spots. A higher frequency of language-positive nTMS spots among more impaired patients according to pre- and postoperative as well as follow-up examinations makes sense as a decline in language function should be associated with higher stimulation-induced errors, although this may bias the results of nTMS language mapping. A previous study is in good accordance with this finding and showed that aphasia as measured by the Berlin Aphasia Score correlated significantly with the incidence of errors during nTMS language mapping; yet, correlations were only evaluated for the preoperative status of language function [51]. Moreover, higher aphasia grading for the postoperative and follow-up status was associated with lower ratios in volume for U-fibers. Hence, this finding may underline the important role of short association fibers for keeping of language function as their impairment, e.g., in the direct perioperative course of tumor resection or related to even subtle perioperative ischemia, could result in language worsening. 

### 4.4. Limitations and Perspectives

When interpreting the results of this study, the following limitations have to be acknowledged. First, the retrospective character and comparatively small sample size restrict the generalizability of the findings. Upcoming studies may include more patients and follow a prospective study design. Second, the finding of lower GMI for language-positive nTMS points when compared to language-negative nTMS points and related interpretation as a potential hint for higher GM density needs further validation. A potential explanation could also be linked to the increased connectivity profile of language-positive nTMS points. Future studies using imaging with higher resolution may provide evidence for our preliminary interpretations. Third, the technique of DTI has its inherent methodological shortcomings, which could lead to aberrant fiber reconstruction and visualization, particularly for crossing or kissing fibers and in the presence of edema, which is commonly observed in relation to brain tumors [52,53]. Other sequences and tracking algorithms are developed to become applicable in the clinical setting, but good alternatives to conventional DTI and its tractography-based analyses are still not available for clinical routine use [54,55]. Fourth, the tractography maps generated by nTMS-based DTI FT for depiction of the language network need further validation, ideally by intraoperative DES as the gold-standard method. In this context, previous studies have evaluated the agreement between preoperative nTMS language mapping and intraoperative DES [13,16,24]; however, on the subcortical level, such correlation analyses in representative samples are largely missing to date. First evidence of associations between preoperative nTMS-based tractography and intraoperative DES results or surgery-related aphasia has been obtained [20,37]. Upcoming studies may use more sophisticated approaches for diffusion-weighted MRI and fiber tractography, ideally combining it with functional data such as nTMS maps after further confirmatory studies. Furthermore, repeated investigations of the language network by serial nTMS language mappings and tractography after surgery may be of interest to track potential plastic effects as well as associations with aphasia grades on a longitudinal scale.

## 5. Conclusions

Language-positive and language-negative areas as determined by nTMS language mapping show differences in cortical and subcortical characteristics among patients diagnosed with GBMs. Specifically, language-positive areas demonstrate lower GMI and an enhanced connectivity profile with higher volume ratios for U-fibers, interhemispheric fibers, and fibers projecting to the cerebellum. While future studies may confirm these results in larger cohorts in the context of a prospective study design, these findings facilitate confidence in the technique of nTMS language mapping and nTMS-based tractography to detect language-involved structures of the human cortico-subcortical language network. 

## Figures and Tables

**Table 1:** Cohort characteristics.

No.	Sex	Age	No. of Language-Positive CPS Sites	No. of Language-Negative CPS Sites	100% FAT for DTI FT with Language-Positive nTMS Points	100% FAT for DTI FT with Language-Negative nTMS Points	Awake Surgery	Aphasia Grading		
								Preop	Postop	Follow-up
1	male	52.0	12	34	0.29	0.32	No	0	0	0
2	male	76.7	18	26	0.32	0.34	No	2	2	1
3	male	68.5	15	30	0.39	0.39	Yes	0	0	0
4	male	54.2	15	31	0.42	0.48	Yes	2	0	0
5	male	52.2	8	38	0.42	0.36	No	1	1	1
6	male	57.0	10	30	0.49	0.58	No	0	0	0
7	female	70.1	12	34	0.29	0.32	No	0	0	0
8	male	64.3	31	15	0.39	0.39	No	3	3	3
9	male	72.6	5	41	0.33	0.38	No	0	0	0
10	male	57.3	8	35	0.29	0.38	Yes	1	1	1
11	male	71.5	35	11	0.33	0.37	Yes	2	2	2
12	male	55.6	28	18	0.39	0.35	No	0	0	0
13	female	72.2	9	35	0.34	0.35	No	1	0	0
14	male	74.4	24	19	0.37	0.36	No	0	2	0
15	male	70.6	16	29	0.37	0.36	No	1	2	1
16	male	62.0	12	33	0.33	0.43	No	0	0	0
17	female	60.5	19	25	0.37	0.43	Yes	2	2	2
18	male	20.3	2	38	0.38	0.44	No	0	0	0
19	female	80.8	5	38	0.30	0.28	No	0	0	0
20	male	70.3	18	28	0.41	0.37	No	2	3	3

This table shows cohort details, including sex distribution and age (in years), information on the number of language-positive and language-negative sites according to the cortical parcellation system (CPS), the fractional anisotropy threshold (FAT) for diffusion tensor imaging fiber tracking (DTI FT) based on navigated transcranial stimulation (nTMS), and aphasia grades (at three different time points: preop = preoperative status, postop = postoperative status, follow-up = status during follow-up examinations three months after surgery). Language mapping aimed to cover 46 target points in total that were placed in relation to the CPS on the left hemisphere (LH), but a reduced number of targets was stimulated in patients with large tumor masses that precluded placement of all 46 target points. Thus, the numbers of language-positive and language-negative CPS sites do not necessarily add up to 46 in all enrolled patients.

**Table 2:** Comparison between language-positive and language-negative mapping and tractography.

Item		Language-Positive nTMS Points		Language-Negative nTMS Points		*p*
		Mean	SD	Mean	SD	
N		15.1	8.9	29.4	8.3	**0.0026**
IR		5.7	1.7	7.1	1.6	**0.0121**
R_Ufibers_	100% FAT	0.3	0.2	0.1	0.1	**0.0012**
	75% FAT	0.5	0.2	0.3	0.3	**0.0020**
	50% FAT	0.6	0.3	0.4	0.2	**0.0056**
	25% FAT	0.7	0.5	0.4	0.3	0.1231
R_cross_		0.9	0.8	0.5	0.6	**0.0494**
R_cereb_		0.6	0.6	0.3	0.5	**0.0094**

This table shows the mean and standard deviation (SD) for the number (N) of language-positive and language-negative points as mapped by navigated transcranial magnetic stimulation (nTMS), intensity ratio (IR), ratio of volumes for U-fibers (R_Ufibers_, as derived from tractography using 100%, 75%, 50%, and 25% of the individual fractional anisotropy threshold (FAT)), and ratio of volumes for interhemispheric fibers (R_cross_, using tractography with 25% FAT) as well as fibers projecting to the cerebellum (R_cereb_, using tractography with 25% FAT). Wilcoxon matched-pairs signed rank tests were conducted to assess differences in these characteristics between language-positive and language-negative mappings (level of statistical significance: *p* < 0.05). Statistically significant values are displayed in bold.

**Table 3:** Associations with aphasia grading.

ROI	Item		Parameter	Aphasia Grading		
				Preoperative	Postoperative	Follow-Up
**Language-Positive nTMS Points**	N		rho	0.4919	0.6183	0.4854
			*p*	**0.0276**	**0.0037**	**0.0300**
	IR		rho	0.0138	−0.0806	0.0888
			*p*	0.9538	0.7354	0.7098
	R_Ufibers_	100% FAT	rho	−0.3777	−0.6102	−0.4899
			*p*	0.1007	**0.0043**	**0.0283**
		75% FAT	rho	−0.2645	−0.4323	−0.4080
			*p*	0.2597	0.0570	0.0741
		50% FAT	rho	−0.0016	0.0590	−0.0854
			*p*	0.9946	0.8048	0.7205
		25% FAT	rho	−0.1823	0.0341	−0.0632
			*p*	0.4417	0.8866	0.7914
	R_cross_		rho	−0.4590	−0.1629	−0.2288
			*p*	0.0418	0.4925	0.3320
	R_cereb_		rho	−0.1717	−0.1347	−0.1110
			*p*	0.4691	0.5713	0.6414
**Language-Negative nTMS Points**	N		rho	−0.4521	−0.6097	−0.4741
			*p*	0.0454	**0.0043**	**0.0347**
	IR		rho	−0.1660	−0.1987	−0.2475
			*p*	0.4842	0.4011	0.2927
	R_Ufibers_	100% FAT	rho	0.0733	−0.1297	−0.0956
			*p*	0.7589	0.5858	0.6885
		75% FAT	rho	−0.2784	−0.2752	−0.3141
			*p*	0.2347	0.2403	0.1774
		50% FAT	rho	−0.1457	0.1621	0.0768
			*p*	0.5400	0.4947	0.7475
		25% FAT	rho	0.1750	0.3267	0.2885
			*p*	0.4606	0.1598	0.2174
	R_cross_		rho	0.0073	−0.0399	0.0598
			*p*	0.9755	0.8674	0.8024
	R_cereb_		rho	0.0887	0.2959	0.1912
			*p*	0.7099	0.2052	0.4194

This table shows the correlation results between the number of language-positive and language-negative points as mapped by navigated transcranial magnetic stimulation (nTMS), intensity ratio (IR), ratio of volumes for U-fibers (R_Ufibers_, as derived from tractography using 100%, 75%, 50%, and 25% of the individual fractional anisotropy threshold (FAT)), ratio of volumes for interhemispheric fibers (R_cross_, using tractography with 25% FAT) as well as fibers projecting to the cerebellum (R_cereb_, using tractography with 25% FAT) and the aphasia grades for the preoperative, postoperative, and follow-up status. Correlation coefficients are represented by Spearman’s rho, and related *p*-values are given (level of statistical significance: *p* < 0.05). Statistically significant values that survived adjustments for multiple testing (Benjamini–Hochberg procedure with a false discovery rate of 25%) are depicted in bold.

## Abbreviations

CPS	Cortical parcellation system
CSF	Cerebrospinal fluid
DES	Direct electrical stimulation
DICOM	Digital Imaging and Communications in Medicine
DTI	Diffusion tensor imaging
DTI FT	Diffusion tensor imaging fiber tracking
EHI	Edinburgh Handedness Inventory
FA	Fractional anisotropy
FAT	Fractional anisotropy threshold
FL	Fiber length
FLAIR	Fluid attenuated inversion recovery
GBM	Glioblastoma multiforme
GM	Gray matter
GMI	Gray matter intensity
GTR	Gross total resection
IR	Intensity ratio
LH	Left hemisphere
MRI	Magnetic resonance imaging
nTMS	Navigated transcranial magnetic stimulation
ROI	Region of interest
SD	Standard deviation
TE	Echo time
TR	Repetition time
